# Burden and correlates of mental health diagnoses among sex workers in an urban setting

**DOI:** 10.1186/s12905-017-0491-y

**Published:** 2017-12-19

**Authors:** Nitasha Puri, Kate Shannon, Paul Nguyen, Shira M. Goldenberg

**Affiliations:** 1 Gender and Sexual Health Initiative, British Columbia Centre for Excellence in HIV/AIDS, St. Paul’s Hospital, 608-1081 Burrard Street, Vancouver, BC V6Z 1Y6 Canada; 2 Department of Family Practice, University of British Columbia, 3rd Floor David Strangway Building, 5950 University Boulevard, Vancouver, BC V6T 1Z3 Canada; 3 School of Population and Public Health, University of British Columbia, 2206 E Mall, Vancouver, BC V6T 1Z3 Canada; 4 Faculty of Health Sciences, Simon Fraser University, 8888 University Drive, Burnaby, BC V5A 1S6 Canada

**Keywords:** Mental health, Trauma, Non-injection drugs, Sexual/gender minority, Women sex workers

## Abstract

**Background:**

Women involved in both street-level and off-street sex work face disproportionate health and social inequities compared to the general population. While much research has focused on HIV and sexually transmitted infections (STIs) among sex workers, there remains a gap in evidence regarding the broader health issues faced by this population, including mental health. Given limited evidence describing the mental health of women in sex work, our objective was to evaluate the burden and correlates of mental health diagnoses among this population in Vancouver, Canada.

**Methods:**

An Evaluation of Sex Workers Health Access (AESHA) is a prospective, community-based cohort of on- and off-street women in sex work in Vancouver, Canada. Participants complete interviewer-administered questionnaires semi-annually. We analyzed the lifetime burden and correlates of self-reported mental health diagnoses using bivariate and multivariable logistic regression.

**Results:**

Among 692 sex workers enrolled between January 2010 and February 2013, 338 (48.8%) reported ever being diagnosed with a mental health issue, with the most common diagnoses being depression (35.1%) and anxiety (19.9%). In multivariable analysis, women with mental health diagnoses were more likely to identify as a sexual/gender minority (LGBTQ) [AOR=2.56, 95% CI: 1.72—3.81], to use non-injection drugs [AOR=1.85, 95% CI: 1.12—3.08], to have experienced childhood physical/sexual trauma [AOR=2.90, 95% CI: 1.89—4.45], and work in informal indoor [AOR=1.94, 95% CI: 1.12 – 3.40] or street/public spaces [AOR=1.76, 95% CI: 1.03–2.99].

**Conclusions:**

This analysis highlights the disproportionate mental health burden experienced by women in sex work, particularly among those identifying as a sexual/gender minority, those who use drugs, and those who work in informal indoor venues and street/public spaces. Evidence-informed interventions tailored to sex workers that address intersections between trauma and mental health should be further explored, alongside policies to foster access to safer workspaces and health services.

## Background

Women involved in sex work face disproportionate social and health inequities compared to the general population, including high rates of violence, poor sexual health, and vulnerabilities to HIV and STIs [1–9]. In comparison to the growing body of research on HIV in sex work, analyses of mental health of women in sex work remain sparse and are limited to only a few studies from urban settings in Europe, North America, Australia and Asia. Evidence from previous studies indicate that women in sex work may experience a high burden of mental illness, especially mood disorder, suicidal ideation, and post-traumatic stress disorder (PTSD) [1–5, 10–13]. While some studies describe associations between mental health and a personal history of trauma, ongoing client or partner violence, and comorbid physical illnesses such as HIV and STIs [4, 14], few studies have explored the links between structural factors and mental health among sex workers. These data remain needed given the importance of interventions which move beyond a sole focus on individual-level risk behaviors for achieving improved population-health outcomes, particularly among marginalized populations such as sex workers [15]. As such, descriptions exploring the associations between structural factors and mental health is needed.

Studies with sex workers in China suggest that individual- and partner-level factors such as sexual coercion, age, and self-stigma may be linked to poorer mental health outcomes, while research from Australia, Mexico, and other U.S. cities (e.g., New York, Miami, San Francisco) indicate links between historical trauma and symptoms of depression, anxiety, and/or PTSD [2, 5, 7, 12, 13, 16, 17]. Studies that examine the mental health of women in sex work who identify as gender/sexual minorities is particularly limited. Themes from Vancouver-based qualitative research examining experiences of transgender sex workers demonstrate that they face serious health and social inequities, including trauma and violence, which are linked to socio-structural factors including transphobia, criminalization, and stigma [18]. Similarly, Nemoto et al. discovered that almost half their population of trans female sex workers in the Bay Area had high depression scores, which were correlated with frequent transphobia experiences and lower income and education [19]. From these early studies, it is apparent that women in sex work are at risk of mental illness that is linked to traumatic experiences, and that the subpopulation of women who identify as sexual/gender minorities may have discrimination related contributors.

Previous research on the health of marginalized populations suggests the critical roles of intersecting individual, interpersonal, and structural factors in shaping health inequities and access to care. The analysis for this study uses an adapted version of our previously published framework on structural determinants of HIV among sex workers [20]. This model examines intersections between individual and interpersonal factors such as condom use, drug use, and socio-demographics that shape sex workers’ sexual health, as well as structural factors including laws, policies and features of work environments. Although limited research has considered structural determinants of mental health among sex workers, prior studies have reported associations between multi-level factors and HIV/STI risks among sex workers [15, 21–26] as well as important relationships between social factors, structural factors and mental health in both general and sex worker populations worldwide [2, 4, 11, 27–29]. For our study, we included individual factors previously demonstrated to be associated with health inequities among sex workers including socio-demographic characteristics such as Indigenous ancestry, sexual/gender minority, and substance use patterns [17, 22, 30, 31]. In addition, we examined common interpersonal/dyad-level factors among sex workers and their clients/partners, including violence, sexual coercion, and influences of drug use [21, 23, 26]. Finally, structural variables including work environment, criminalization/policing, unstable housing, and trauma were examined based on previous studies [15, 20, 26, 32–36]. We hypothesized that our adapted framework will demonstrate correlations between mental illness and structural factors, as well as the individual and interpersonal factors already documented in the literature as described above.

This study was conducted in Metropolitan Vancouver, Canada, where sex workers come from diverse backgrounds, encompassing a range of ages, sexual orientations, education, income levels, and gender expression, although the majority identify as women [6]. Sex work takes place in private venues such as escort agencies, massage parlours, hotels/motels, private residences, as well as informal settings such as bars, motels, and on the streets [1]. Certain sectors of the industry in Canada are highly racialized, with women of Indigenous ancestry disproportionally represented in street-level sex work [6, 22, 37]. This stems from a history of colonial and racialized policies and practices that resulted in the displacement, dispossession, and marginalization of Indigenous peoples.

Given limited evidence on sex workers’ mental health in a Canadian setting, we aimed to evaluate the burden and correlates of having a mental health diagnosis among on- and off-street sex workers in Metropolitan Vancouver, Canada, using an adapted structural determinants framework. This research remains an important first step for beginning to elucidate mental health inequities and potential intervention approaches for this population.

## Methods

### Study Design

Baseline data was drawn from An Evaluation of Sex Workers Health Access (AESHA), an open prospective cohort study. As part of our open prospective design, sex workers in the cohort are followed every six months, and new participants are recruited annually to address attrition and maintain a large sample under continuous follow-up. Between January 2010 and February 2013, 692 female sex workers were enrolled and completed surveys and biological testing for HIV, sexually transmitted infections (STIs), and hepatitis C (HCV). The AESHA study was developed based on substantial community collaborations with sex work agencies since 2005 [38] and was monitored by a Community Advisory Board of representatives of 15+ community agencies. The study holds ethical approval through Providence Health Care/University of British Columbia Research Ethics Board.

As previously described [39], eligibility for the study includes self-identifying as a woman/female, including transgender individuals who identify as women (male-to-female); having exchanged sex for money within the last 30 days; and providing written informed consent. Time-location sampling was used to recruit participants in order to accommodate the challenges of recruiting sex workers who are in isolated and hidden locations, and for whom there is no census. Time location sampling is a probability-based sampling method that allows for recruitment of members of hidden populations at the times and places where they are most likely to congregate [40]. Through outreach to diverse on-street (e.g. streets, alleys) and off-street settings (e.g. online, newspapers, massage parlours, micro-brothels, and in-call locations) across Metro Vancouver, community mapping was done with current/former sex workers who identified and updated venues that were used to identify times and places for our study recruitment [38].

At enrolment and 6-month follow-up intervals, sex workers responded to a questionnaire administered by a trained interviewer (including experiential staff and staff with extensive community experience) and were offered voluntary pre/post-test counseling and HIV/STI/HCV serology testing by a project nurse [39]. Mental health measures included in the questionnaire were developed based on community and sex worker input, and gleaned information about self-reported mental health diagnoses (e.g., depression, PTSD) and access to care for mental illness. All participants received an honorarium of $40CAD at each bi-annual visit for their time, expertise and travel. All participants received post-test counseling, and nursing staff provided referral and active connections to service providers (e.g., mental health services, HIV providers, STI treatment).

### Statistical Analyses

The dependent variable – self-reported mental health diagnoses – was defined as ever having been diagnosed with a mental health condition during the participant’s lifetime. This included responding ‘yes’ to any of the following diagnoses at baseline: depression; post-traumatic stress disorder (PTSD); anxiety; schizophrenia; borderline personality disorder; attention deficit hyperactivity disorder (ADHD); bipolar disorder; and other diagnosis specified.

Independent variables hypothesized to be associated with mental health diagnosis were selected a priori. Variable selection was guided by previous literature on mental health, both in the general population [27, 41] and the sex worker population [4, 7, 28, 33, 42]. This previous literature indicates the importance of individual factors such as education, race and ethnicity, and gender/sexual minority status and power dynamics; interpersonal factors such as sexual and drug risks; and structural factors including poverty, violence, working conditions, and physical environments in shaping the mental health of various populations.

Of interest, independent variables of significance were defined based on questionnaire responses. Indigenous was defined as ‘yes’ to any of ‘First Nations’ or ‘Métis’ or ‘Inuit’. Sexual/gender minority was defined as any of ‘gay’, ‘lesbian’, ‘bisexual’, ‘transgender’, ‘transsexual’, ‘two-spirit’, or ‘gender diverse’ as compared to cisgender and straight. Illicit non-injection drug use, excluding cannibas, and illicit injection drug use were defined as ‘yes’ to using a list of illicit substances including heroin, cocaine, crack, crystal methamphetamine, prescription opioids, benzodiazepines, illicit methadone, and others at baseline. Childhood abuse was defined as ‘yes’ to ever been physically assaulted, touched sexually, or made to do something sexual against their will before the age of 18. Regarding work environments, primary place of service was coded as outdoor/public spaces (street, vehicle, other public areas); informal indoor establishment (crack/drug houses, bars, nightclubs, hotels, client’s place, worker’s place, and housing); or formal indoor establishment (massage parlours, health/beauty enhancement centers, and micro-brothels).

Correlates of lifetime mental health diagnoses at baseline were examined using bivariate and multivariable logistic regression. The differences in the characteristics between those who reported a mental health diagnosis and those who did not were assessed using the Mann-Whitney test for continuous variables and Pearson’s chi-squared test (or Fisher’s exact test for small cell counts) for categorical variables. Variables that were a priori hypothesized to be related to mental health diagnosis and with a significance level of *p*<0.10 in bivariate analyses were considered for inclusion in the multivariable analysis. Model selection was performed using a backwards selection approach. The Akaike information criterion (AIC) was used to determine the most parsimonious model, as indicated by the lowest AIC value (824.703 in our model). Analyses were performed using the SAS software version 9.3 (SAS, Cary, NC). All tests of significance were two-sided, and a *p*-value of 0.05 or less was selected for defining statistical significance.

## Results

Among 692 sex workers, 338 (48.8%) reported having ever been diagnosed with a mental health condition. Depression was the most common self-reported mental health diagnosis (35.1%), followed by anxiety (19.9%), post-traumatic stress disorder (PTSD) (12.7%), and bipolar disorder (10.3%). Less commonly reported were attention deficit hyperactivity disorder (ADHD) (4.9%), borderline personality disorder (3.5%) and schizophrenia (2.3%). Of the 338 sex workers who reported mental health diagnosis, 273 (80.8%) had received treatment and/or counseling in their lifetime.

One third (36.3%) of women were Indigenous, 25.6% identified as a sexual/gender minority, and 11.3% were living with HIV (Table 1). Forty percent (40.0%) reported using injection drugs, 69.4% reported non-injection drug use, and 30.1% exchanged sex directly for drugs at baseline. Intimate partner violence was experienced by 21.2% of sex workers, and 23.4% reported client physical or sexual violence. The majority of sex workers (71.2%) reported childhood (i.e., when age <18 years) physical or sexual abuse. Primary places of service included outdoor/public spaces (44.4%), informal indoor spaces (25.7%) and formal indoor establishments/brothels (29.9%). Police harassment was reported by 40%, and 30.8% had experienced homelessness. At baseline, the median age was 34 (interquartile range [IQR]: 28-41), with no significant differences based on reported mental health diagnoses.

**Table 1:** Baseline characteristics of women sex workers in Vancouver, Canada, 2010–2013 (*n*=692), stratified by mental health diagnoses

Characteristic	Total (*n*=692) n (%)	Yes MH (*n*=338) n (%)	No MH (*n*=354) n (%)	*p*-value
Individual Biological and Behavioural Factors				
Age (median, IQR)	34 (28 – 42)	34 (28 – 42)	35 (28 – 41)	0.434
Indigenous ancestry	251 (36.3)	152 (45.0)	99 (28.0)	<0.001
Sexual/gender minority^a^	177 (25.6)	128 (37.9)	49 (13.8)	<0.001
Injection drug use^b^	277 (40.0)	173 (51.2)	104 (29.4)	<0.001
Non-injection drug use^b^	480 (69.4)	288 (85.2)	192 (54.2)	<0.001
High school attainment or greater	361 (52.2)	155 (45.9)	206 (58.2)	0.001
HIV positive	78 (11.3)	41 (12.1)	37 (10.5)	0.485
STI positive	77 (11.1)	37 (11.0)	40 (11.3)	0.883
Partner/Interpersonal Risks				
Injection use by partner^b^	69 (10.0)	49 (14.5)	20 (5.7)	<0.001
Non-injection use by partner^b^	242 (35.0)	143 (42.3)	99 (28.0)	<0.001
Inconsistent condom use by clients^b^	124 (17.9)	86 (25.4)	38 (10.7)	<0.001
Having sex while high^b^	425 (61.4)	257 (76.0)	168 (47.5)	<0.001
Exchanging sex for drugs^b^	208 (30.1)	130 (38.5)	78 (22.0)	<0.001
Physical/sexual violence by intimate partner^b^	147 (21.2)	90 (26.6)	57 (16.1)	0.001
Physical/sexual violence by clients^b^	162 (23.4)	109 (32.3)	53 (15.0)	<0.001
Structural Determinants				
Recent homelessness^b^	213 (30.8)	128 (37.9)	85 (24.0)	<0.001
Childhood trauma^c^	493 (71.2)	294 (87.0)	199 (56.2)	<0.001
Police harassment^b^	277 (40.0)	159 (47.0)	118 (33.3)	<0.001
Primary place of service^b^				
Formal indoor establishment/brothel	207 (29.9)	48 (14.2)	159 (44.9)	
Outdoor/public space	307 (44.4)	180 (53.3)	127 (35.9)	<0.001
Informal indoor establishment	178 (25.7)	110 (32.5)	68 (19.2)	<0.001

^a^Defined as ‘yes’ to any of ‘gay’, ‘lesbian’, ‘bisexual’, ‘transgender’, ‘transsexual’, ‘two-spirited’, ‘other’

^b^In the last six months

^c^Physical or sexual assault before age 18

MH – mental health; *p*-value reported for bivariate correlation between MH yes and variable, *p*<0.05 for inclusion in multivariate model

In bivariate analyses (Table 2), most variables were significant, and sex workers with a mental health diagnosis were more likely to be of Indigenous ancestry [odds ratio (OR)=2.11, 95% confidence interval (CI): 1.54-2.89], identify as a sexual/gender minority [OR=3.79, 95% CI: 2.61-5.51], to have used non-injection drugs [OR=4.86, 95% CI: 3.37-7.01] or exchanged sex for drugs [OR=2.21, 95% CI: 1.58-3.09]. In addition, interpersonal factors such as inconsistent condom use [OR=2.84, 95% CI: 1.87-4.30], exchanging sex while high [OR=3.51, 95% CI: 2.53-4.86], and violence by clients [OR=2.70, 95% CI: 1.87-3.92] were significantly associated with a greater burden of mental health diagnoses. In terms of structural determinants, historical childhood physical/sexual abuse [OR=5.20, 95% CI: 3.56-7.61] was positively correlated with mental health diagnoses, while working in outdoor/public spaces and informal indoor spaces (vs. formal indoor places) was associated with a higher burden of mental health diagnoses [OR=0.21, 95% CI: 0.14-0.32].

**Table 2:** Bivariate and multivariable analyses of factors correlated with mental health diagnoses among sex workers in Vancouver, Canada, 2010–2013 (*n*=692)

Characteristic	Unadjusted Odds Ratio (95% CI)	*p*-value	Adjusted Odds Ratio (95% CI)	*p*-value
Individual Biological and Behavioural Factors				
Indigenous ancestry	2.11 (1.54 – 2.89)	<0.001		
Sexual/gender minority^a^	3.79 (2.61 – 5.51)	<0.001	2.56 (1.72 – 3.81)	<0.001
High school attainment or greater	0.61 (0.45 – 0.82)	0.001		
Non-injection drug use^b^	4.86 (3.37 – 7.01)	<0.001	1.85 (1.12 – 3.08)	0.017
Injection drug use^b^	2.52 (1.84 – 3.45)	<0.001		
Partner/Interpersonal Risks				
Inconsistent condom use^b^	2.84 (1.87 – 4.30)	<0.001		
Having sex while high^b^	3.51 (2.54 – 4.86)	<0.001		
Exchanging sex for drugs^b^	2.21 (1.58 – 3.09)	<0.001		
Physical/sexual violence by intimate partner^b^	1.89 (1.30 – 2.74)	0.001		
Physical/sexual violence by client^b^	2.70 (1.87 – 3.92)	<0.001		
Structural Determinants				
Recent homelessness^b^	1.93 (1.39 – 2.68)	<0.001		
Childhood trauma^v^	5.20 (3.56 – 7.61)	<0.001	2.90 (1.89 – 4.45)	<0.001
Police harassment^b^	1.78 (1.31 – 2.42)	<0.001		
Primary place of service^b^				
Formal indoor establishment/brothel (Reference)				
Informal indoor establishment	5.36 (3.44 – 8.34)	<0.001	1.94 (1.11 – 3.40)	0.021
Outdoor/public space	4.70 (3.16 – 6.97)	<0.001	1.76 (1.03 – 2.99)	0.038

^a^Defined as ‘yes’ to any of ‘gay’, ‘lesbian’, ‘bisexual’, ‘transgender’, ‘transsexual’, ‘two-spirited’, ‘other’

^b^In the last six months

^c^Physical or sexual assault before age 18

MH – mental health; *p*-value reported for bivariate correlation between MH yes and variable, *p*<0.05 for inclusion in multivariate model

In multivariable analysis (Table 2), sex workers with mental health diagnoses were more likely to identify as a sexual/gender minority (LGBTQ) [adjusted odds ratio (AOR)=2.56, 95% CI: 1.72-3.81], ever used non-injection drugs [AOR=1.85, 95% CI: 1.12-3.08], historical childhood physical/sexual abuse [AOR=2.90, 95% CI: 1.89-4.45], and work in outdoor/public spaces [AOR=1.76, 95% CI: 1.03-2.99] or informal indoor establishments [AOR=1.94, 95% CI: 1.12-3.40].

## Discussion

Sex workers with mental health diagnoses were more likely to identify as a sexual/gender minority, use non-injection drugs, have experienced childhood trauma, and work in informal indoor venues or street/public spaces. This study is one of the first in Canada that quantitatively examines the burden of mental health diagnosis among sex workers in an urban setting, and contributes to current evidence exploring multilevel correlates of disease burden.

This study documented a significantly higher burden of mental health diagnosis among sex workers who work in street and informal indoor venues (such as bars and hotels) as compared to formal sex work establishments (in-call spaces). While safer work environments have been linked to enhanced HIV/STI prevention and lower prevalence of violence and criminalization [21, 35, 43, 44], work environments have rarely been studied in relation to sex workers’ mental health [45]. Although we cannot determine causal relationships between lifetime mental health diagnosis and place of work at the time of the study, we can surmise possible contributors to mental health burden with respect to place of work. In studies from Australia and San Francisco, mental health issues have been linked to street level work environments [45, 46], which authors suggest is due to vulnerability from doing sex work illegally in unsanctioned spaces. In addition, some research suggests that high levels of burnout in informal indoor workplaces may be linked to greater burden of mental health diagnoses (e.g., depression, anxiety) [47]. In Vancouver, the street sex scene overlaps substantially between the city’s drug scene [48]; perhaps the (non-injection) drug use in this setting contributes to our findings of mental health diagnosis burden in this place of service. Given that mental health diagnoses appear to be concentrated among sex workers in street and informal indoor settings in our study, and in light of previous evidence highlighting the multitude of structural risks faced by sex workers in Vancouver (e.g. criminalization, police harassment, violence and reduced control over sexual negotiation) [21, 35, 43, 44], the consideration and development of legislative changes that ensure non-criminalization and improve access to safer, formal indoor work spaces remains needed in this setting.

Similar to our results, previous studies from U.S., Australian, and Latin American cities also suggest that traumatic experiences (both in early childhood and in adulthood while engaging in sex work) contribute to mental illness among sex workers [4, 5, 10, 12, 49]. For example, in Sydney, Australia, 47% of sex workers met DSM-IV criteria for PTSD, and 31% of the sample (67% of those with PTSD) reported current symptoms; the majority had also experienced historical trauma, most often in childhood [10]. For Indigenous women especially, who are overrepresented in our sample as well as the most visible aspects of Canada’s sex industry, high rates of childhood trauma are deeply rooted in history of colonization and intergenerational trauma [50–54], including the significant emotional, physical and sexual abuse faced by Indigenous children exposed to Canada’s residential school system [24, 55, 56]. As such, trauma-informed mental health care practices may be further tailored to the unique needs of sex workers (in particular, Indigenous sex workers), within a decolonizing and self-determinant framework [43].

Our study also uniquely documented linkages between identifying as a person of sexual/gender minority (such as trans, lesbian, gay, bisexual, two spirited, or gender diverse) and having a mental health diagnosis among sex workers in an urban Canadian context. These results may be explained by the complex interplay between structural determinants such as stigma, trauma, discrimination and transphobia, and high levels of violence and assault faced by these populations [19, 57–60]. Nemoto et al.’s study further describes the post-traumatic effects of physical violence in this population, and the stark lack of social supports and help-seeking behaviour by trans sex workers due to fear of police or health care providers [19]. Research indicates that high rates of community and internalized (self-) stigma among sexual/gender minority people may contribute to a higher burden of mental health problems in this population [57, 61, 62]. In another U.S. study, sexual/gender minority individuals were found to demonstrate significantly higher levels of acute stress and general anxiety than other groups, and were more likely to be victims of sexual assault than non-sexual/gender minorities in an emergency department [58]. Finally, a Vancouver-based study of LGBT sex workers identified similar correlates of client perpetrated violence [60], again highlighting the underlying theme of trauma in this population.

Our results indicate the disproportionate burden of mental health diagnoses given to sex workers who use drugs. Previous studies have highlighted the potential for health disparities experienced by marginalized populations (including sex workers) to frequently overlap, including co-morbidities such as HIV, substance use, and poor mental health [1, 5, 6, 11, 63, 64]. Furthermore, two Vancouver-based studies also highlight the correlations between trauma and substance use, showing that prescription non-opiate use among sex workers and crystal methamphetamine use in youth are associated with increased violence [31, 65]. Although not well documented in the literature, it is possible that mental health symptoms may in some cases resemble withdrawal symptoms, creating challenges when disentangling mental health diagnostics from broader harm reduction initiatives.

These results of high burden of mental health diagnoses among women in sex work and its linkages to various forms of trauma underscore an urgent need to further explore trauma-informed care and practice, including clinical training and system wide policies that adopt resiliency perspectives and address intersections between historical colonization, stigma/discrimination, policing, and substance use [52, 53, 66]. An existing model that is pioneering peer-based mental health treatment paired with advocacy and physical and sexual health care is the St. James Infirmary in San Francisco, California. Research from this organization shows that many sex workers fear stigma from health care providers, and as such interventions offering peer support, safe spaces, and collective organizing capacity remain critical for successfully achieving improved health and social outcomes for sex workers [7]. Further studies are also warranted to better understand and address the diverse mental illness experiences and needs of women in sex work who are operating across a range of work environments and urban settings. Community-based and mixed-methods approaches which explore relationships between structural, historical, individual and interpersonal factors are necessary to inform tailored, trauma-informed interventions that better address the complex and overlapping correlates of depression, PTSD, and anxiety.

*Limitations:* The main outcome of this analysis was self-reported mental health diagnoses at baseline. Although participants are offered direct referral to mental health services, this measure was not based on a formal assessment of mental health symptomatology. In addition, we acknowledge that cross-sectional studies are limited in their capacity to imply causal associations, and recognize that our logistic regression methods offer correlations rather than clearly defined causality. That said, given limited research on sex workers’ mental health, we feel that this study remains an important first step for beginning to elucidate mental health inequities and potential intervention approaches for gender/sexual minorities, women who use drugs, and sex workers in informal and street-based settings. While there is potential for the under-reporting of sensitive and stigmatized behaviors and experiences (e.g., trauma, substance use), our frontline staff (which includes experiential staff) possess extensive community experience, engage in regular outreach, maintain high levels of rapport with participants, and are highly skilled in non-judgmental interviewing techniques, which in our experience are highly successful strategies for creating a safe and non-judgmental research environment to promote accurate responses. In addition, to enable our capacity to assess potential differences experienced by gender/sexual minority groups, our analysis included both cis- and transgender sex workers; we acknowledge that larger studies with the capacity to further elucidate the unique experiences of gender/sexual minorities involved in sex work is critically needed.

## Conclusions

In conclusion, this study highlights the disproportionate burden of mental health burden faced by women in sex work from Metropolitan Vancouver, especially among those who use drugs, identify as a sexual/gender minority, and have a history of childhood trauma. In addition, it elucidates the disproportionate burden of mental health diagnoses among sex workers who work in informal and outdoor spaces, suggesting the need to further explore appropriate outreach and safer workplace interventions to support sex workers’ mental health. Further research that explores mental health screening, diagnosis, and treatment for these vulnerable subpopulations is needed in order to develop evidence-informed interventions.

## Abbreviations

ADHD: Attention Deficit Hyperactivity Disorder
AESHA: An Evaluation of Sex Workers Health Access
AIC: Akaike Information Criterion
AOR: Adjusted Odds Ratio
CI: Confidence Interval
DSM IV: Diagnostic and Statistical Manual of Mental Disorders IV
HCV: Hepatitis C Virus
HIV: Human Immunodeficiency Virus
HSV: Herpes Simplex Virus
IQR: Interquartile Range
LGBTQ: Lesbian, Gay, Bisexual, Trans, Queer
OR: Odds Ratio
PTSD: Post Traumatic Stress Disorder
STI: Sexually Transmitted Infection
US: United States
